# Protocatechuic Acid, a Phenolic from *Sansevieria roxburghiana* Leaves, Suppresses Diabetic Cardiomyopathy via Stimulating Glucose Metabolism, Ameliorating Oxidative Stress, and Inhibiting Inflammation

**DOI:** 10.3389/fphar.2017.00251

**Published:** 2017-05-08

**Authors:** Niloy Bhattacharjee, Tarun K. Dua, Ritu Khanra, Swarnalata Joardar, Ashis Nandy, Achintya Saha, Vincenzo De Feo, Saikat Dewanjee

**Affiliations:** ^1^Advanced Pharmacognosy Research Laboratory, Department of Pharmaceutical Technology, Jadavpur UniversityKolkata, India; ^2^Department of Chemical Technology, University of CalcuttaKolkata, India; ^3^Department of Pharmacy, University of SalernoSalerno, Italy

**Keywords:** diabetic cardiomyopathy, glucose utilization, inflammation, oxidative stress, protocatechuic acid, *Sansevieria roxburghiana*, type 2 diabetes mellitus

## Abstract

Persistent hyperglycemia, impairment of redox status and establishment of inflammatory pathophysiology integrally play important role in the pathogenesis of diabetic cardiomyopathy (DC). Present study examined the therapeutic potential of protocatechuic acid isolated from the *Sansevieria roxburghiana* rhizomes against DC employing rodent model of type 2 diabetes (T2D). T2D was induced by high fat diet + a low-single dose of streptozotocin (35 mg/kg, i.p.). T2D rats exhibited significantly (*p* < 0.01) high fasting blood glucose level. Alteration in serum lipid profile (*p* < 0.01) and increased levels of lactate dehydrogenase (*p* < 0.01) and creatine kinase (*p* < 0.01) in the sera of T2D rats revealed the occurrence of hyperlipidemia and diabetic pathophysiology. A significantly (*p* < 0.01) high levels of serum C-reactive protein and pro-inflammatory mediators revealed the establishment of inflammatory occurrence in T2D rats. Besides, significantly high levels of troponins in the sera revealed the establishment of cardiac dysfunctions in T2D rats. However, protocatechuic acid (50 and 100 mg/kg, p.o.) treatment could significantly reverse the changes in serum biochemical parameters related to cardiac dysfunctions. Molecular mechanism studies demonstrated impairment of signaling cascade, IRS1/PI3K/Akt/AMPK/p 38/GLUT4, in glucose metabolism in the skeletal muscle of T2D rats. Significant (*p* < 0.01) activation of polyol pathway, enhanced production of AGEs, oxidative stress and up-regulation of inflammatory signaling cascades (PKC/NF-κB/PARP) were observed in the myocardial tissue of T2D rats. However, protocatechuic acid (50 and 100 mg/kg, p.o.) treatment could significantly (*p* < 0.05–0.01) stimulate glucose metabolism in skeletal muscle, regulated glycemic and lipid status, reduced the secretion of pro-inflammatory cytokines, and restored the myocardial physiology in T2D rats near to normalcy. Histological assessments were also in agreement with the above findings. *In silico* molecular docking study again supported the interactions of protocatechuic acid with different signaling molecules, PI3K, IRS, Akt, AMPK PKC, NF-κB and PARP, involved in glucose utilization and inflammatory pathophysiology. *In silico* ADME study predicted that protocatechuic acid would support the drug-likeness character. Combining all, results would suggest a possibility of protocatechuic acid to be a new therapeutic agent for DC in future.

## Introduction

Diabetes mellitus (DM) is a chronic metabolic syndrome has climbed markedly over past few decades (World Health Organization [WHO], 2016). The global prevalence of DM has risen to 8.5% amounting ∼422 million in 2014 (World Health Organization [WHO], 2016). It has been predicted that, the incidence of this syndrome would be more than double by the year of 2030 (Fernández-Millán et al., 2014). Amongst all diagnosed cases, type 2 diabetes mellitus (T2DM) is more rampant and comprising ∼90–95% of total diabetic cases (Khanra et al., 2015). Hyperglycemia arbitrated glucose toxicity resulted a number of homeostatic disturbances within the organs, which results a number of complications in the critical organs. Diabetic cardiomyopathy (DC) is one of the major complications in T2DM. It has been reported that, adults with T2DM historically have 2–3 times higher risk of cardiovascular problems than the adults without T2DM (World Health Organization [WHO], 2016). Persistent hyperglycemia introduces toxic effects through a sequence of secondary transducers. The excess generation of reactive oxygen species (ROS) in the myocardial tissue via activation of polyol pathway is considered to be one of the principle mechanisms in the development of DC. Glucose oxidation resulted production of excess of advanced glycation end-products (AGEs) which leads to myocardial inflammation, collagen deposition and fibrosis (Riaz et al., 2016). AGEs and oxidative stress have been reported to activate nuclear factor kappa-beta (NF-κB) and protein kinase C (PKC) signaling and thereby induce inflammation (Tsai and Yin, 2012; Bhattacharjee et al., 2016a). Considering the involvements of multiple toxicological events in DC, the attenuation of these events in diabetic population is the looming issue in the field of clinical diabetology. Increased glucose metabolism, i.e., glycemic control is one of the principle means to control DC. Reduction of inflammation, oxidative stress and polyol enzymes would further contribute in attenuation of DC. Induction of a sequence of secondary transducers due to persistent hyperglycemia is principally responsible for the development and progression of DC. Therefore, glycemic control would be the principle intention to control DC. Besides hypoglycemic effect, anti-oxidant and anti-inflammatory effects would potentiate the therapeutic efficacy of a therapeutic agent in DC.

*Sansevieria roxburghiana* Schult. & Schult. F. (Family: Asparagaceae) is a perennial herb with fleshy stem and enviable rootstock. The crude extract of the rhizomes of this plant has been reported to possess prophylactic effect against DC by our group (Bhattacharjee et al., 2016b). Protocatechuic acid has been isolated from the rhizomes of *S. roxburghiana*. Protocatechuic acid has been reported to possess antioxidant and anti-inflammatory effect (Tsai and Yin, 2012; Adefegha et al., 2016; Erukainure et al., 2017). Protocatechuic acid has also been reported to possess hypoglycemic activity against experimentally induced type I diabetic rats without effecting the glycemic status of normal rats (Harini and Pugalendi, 2010). However, mechanism of hypoglycemic effect is yet to be explored. Considering the ethnomedicinal evidences of *S. roxburghiana* rhizomes coupled with reported pharmacological effects of protocatechuic acid, present study was undertaken to evaluate the therapeutic benefit of protocatechuic acid against DC in T2D rats. It has been aimed to explore the mechanism of action. Finally, *in silico* molecular docking and ADME studies were performed to demonstrate the probable interactions between protocatechuic acid with signal proteins and the possible safety profile of the same compound.

## Materials and Methods

### Chemicals

Streptozotocin was procured from Hi-media (Mumbai, India). Protocatechuic acid (≥97%), Bradford reagent and bovine serum albumin were procured from Sigma-Aldrich (St. Louis, MO, USA). The antibodies were purchased from Santa Cruz Biotechnology (Santa Cruz, CA, USA) and Sigma-Aldrich (St. Louis, MO, USA). The kits/reagents for biochemical assays for estimating different biochemical parameters were bought from Span diagnostic, Ltd, India and Sigma-Aldrich, USA. All other reagents, solvents, and chemicals used were of analytical grade.

### Extraction of Protocatechuic Acid

The powdered rhizomes of *S. roxburghiana* were macerated with methanol with constant stirring. The crude extract was fractioned with *n*-hexane using separating funnel. The residue was chromatographed in a normal phase silica gel column and eluted with *n-*hexane-ethyl acetate and ethyl acetate-methanol with increasing polarity, to yield eight major fractions (A-H). Fraction E was further chromatographed with *n*-hexane-CH_2_Cl_2_ and CH_2_Cl_2_-methanol with increasing polarity to yield four sub-fractions (E_1-4_). The sub-fraction E_3_ was further column chromatographed using same solvent system and finally purified by preparative TLC using solvent system CH_2_Cl_2_: acetone: acetic acid (10: 1.5: 0.5, v/v/v) to yield protocatechuic acid (1.9% w/w). The structure has been elucidated employing ^1^H and ^13^C NMR interpretation and mass spectroscopic data (Martin et al., 2000; Gutzeit et al., 2007).

### Animals and Diet

Wistar rats (♂, 150 ± 20 g) were housed in separate polyprophylene cages under standard laboratory conditions of temperature (24 ± 2°C), relative humidity (55 ± 5%), light:dark schedule (12 h:12 h), standard rat diet (Agro Corporation Private, Ltd, Bangalore, India) and water *ad libitum* (Dewanjee et al., 2009). The experiment was performed at the animal house (Registration No. 0367/01/C/CPCSEA, UGC, India) of the Department of Pharmaceutical Technology, Jadavpur University, India. The animal experiment has been permitted by the Jadavpur University animal ethical committee (Ref no. AEC/PHARM/1501/01/2015 dated 18.03.2015) and the principles of laboratory animals care were followed during experiment (Public Health Service [PHS], 1986). The animals were acclimatized for 2 weeks before the execution of the *in vivo* experiment.

### Experimental Scheme

High fat fed-low single dose of streptozotocin model for T2DM was used in this study (Reed et al., 2000; Srinivasan et al., 2004). Briefly, the Wistar rats were fed high fat diet (Bhattacharjee et al., 2016b) *ad libitum* for 2 weeks. After 2 weeks, the rats were injected a single dose of streptozotocin (35 mg/kg body weight, i.p.). 1 week after streptozotocin treatment, the rats exhibited fasting blood glucose levels 170 ± 30 mg/dl were considered to be type 2 diabetic (T2D) rats and included for the further experiments. This experimental model for T2D was validated by our group (Bhattacharjee et al., 2016b). The rats were continued with high fat diet throughout the course of the study. One group of normal rats receiving normal diet was kept as normal control.

The rats were divided into three groups (*n* = 6) and received the treatment as follows:

Group I: Normal control rats were administered distilled water (2 ml/kg body weight, p.o.) daily for 28 days;
Group II: T2DM control rats were administered high fat diet + distilled water (2 ml/kg body weight, p.o.) daily for 28 days;
Group III: T2D rats were administered high fat diet + protocatechuic acid (50 mg/kg body weight, p.o.) daily for 28 days;
Group IV: T2D rats were administered high fat diet + protocatechuic acid (100 mg/kg body weight, p.o.) daily for 28 days.

The experimental rats were fasted overnight and the fasting blood glucose levels were measured on days 0, 1, 3, 7, 14, 21, and 28 using single touch glucometer (Ascensia Entrust, Bayer Health Care, USA) (Dewanjee et al., 2009). The body weights, food intake and water intake were monitored in aforementioned time table. After the last treatment, animals were fasted overnight and the blood samples were withdrawn from retro-orbital venous plexus after applying tetracaine (0.5%) ophthalmic drop to the eye of rats (Dua et al., 2016b). The animals were euthanatized and the hearts were excised, cleaned immediately with cold phosphate buffer saline (pH 7.4) (Dua et al., 2016a). The hearts were immediately subjected to different processing for histological, biochemical and immunoblotting analyses. In search of mechanism of hypoglycemic effect, the soleus muscle (skeletal muscle) were collected from individual rats under different treatments and subjected to lysis.

### Estimation of Serum Biochemical Parameters

Serum insulin level was measured by ELISA using commercially available kit (Sigma-Aldrich, USA). Homeostatic model assessments were performed by estimating HOMA-IR and HOMA-β using the following equations (Bhattacharjee et al., 2016b):

HOMA-IR=(Fasting serum insulin in U/1×Fasting blood glucose in mmol/1)/22.5

HOMA-β=(Fasting serum insulin in U/1×20/Fasting blood glucose in mmol/1)−3.5

The serum lipid profile viz. total cholesterol, HDL cholesterol, and triglycerides levels were estimated by commercially available kits (Span Diagnostic, Ltd, India) following manufacturer’s instructions. LDL cholesterol level was estimated following Friedewald’s equation, LDL cholesterol = Total cholesterol – Triglycerides/5 – HDL cholesterol (Friedewald et al., 1972). Serum lactate dehydrogenase (LDH) and creatinin kinase (CK) levels were estimated by the commercially available kits (Span Diagnostic, Ltd, India) following manufacturer’s instructions. Glycosylated hemoglobin concentration was estimated according to the protocol described by Nayak and Pattabiraman (1981). The AGEs in the sera were measured by ELISA (Abcam, Cambridge, UK) as per the manufacturer’s instructions. Troponin I and T contents were determined by ELISA kits (Kamiya Biomedical Company, USA). IL 1β, IL 6, IL 12 and TNF α levels in the sera were measured by ELISA kits (Fisher Thermo Scientific, Co., USA).

### Western Blotting of Signal Proteins in Skeletal Muscle

The soleus muscles of the rats of different groups were homogenized with ice cold lysis buffer. Proteins in the subcellular fractions were obtained by density gradient centrifugation method (Thurmond et al., 1998). Protein samples were quantified by ELISA (Bio-Rad, USA). The protein samples (20 μg) were subjected to SDS-polyacrylamide gel (12%) electrophoresis and western blotted as described by Dewanjee et al. (2015). Briefly, the separated proteins in gel were transferred into nitrocellulose membrane. The membranes were blocked for 1 h at room temperature by treating with blocking buffer (containing 5% non-fat dry milk). The membranes were then incubated with primary antibodies at 4°C overnight followed by washing with tris-buffered saline (TBST). The membranes were then subjected to suitable HRP-conjugated secondary antibody at room temperature for 1 h. The blots were finally documented by 3, 3′ -diaminobenzidine tetrahydrochloride (Bangalore Genei, India). The membranes were then subjected to mild stripping using stripping buffer containing 1% SDS (pH 2.0) and glycine (25 mM) followed by treatments with primary and secondary antibodies for detecting the expressions of other proteins in a single membrane. The expressions of phospho-IRS-1(Tyr 895), PI3K (p85), phospho-Akt (Ser 473), phospho-AMPK (Thr 172) and phospho-P38 (Tyr 180/Tyr 182) were studied. Normalization of the expressions of proteins was done by using GAPDH as loading control.

### Estimation of Biochemical Parameters of Myocardial Tissues

The cardiomyocytes were isolated from the immediately decapitated hearts of the experimental rats following the method described by Nair and Nair (1997) with little modification (Raghu and Cherian, 2009). Briefly, decapitated hearts were rapidly immersed in cold Ca^2+^-free solution. The ventricular myocardial cells were isolated using Langendorff apparatus employing retrograde perfusion through the aorta with enzyme-containing solutions. Finally, Ca^2+^-replication was done to obtain Ca^2+^-tolerant cardiomyocytes. Intracellular ROS production was performed in accordance to the method of LeBel and Bondy (1990) employing 2,7-dichlorofluorescein diacetate (DCF) as a probe. The hearts were homogenized in 0.1 M Tris-HCl-0.001 M EDTA buffer (pH 7.4) and centrifuged (@ 12,000 *g*; 30 min; 4°C). The supernatants were collected for the biochemical assays. The extent of lipid peroxidation (thiobarbituric acid reactive substances, TBARS, by-products of lipid peroxidation) was estimated following the method of Ohkawa et al. (1979). The carbonylation of proteins was measured as per the method described by Uchida and Stadtman (1993). Co-enzymes Q_9_ and Q_10_ were appraised employing RP-HPLC as per standard protocol (Zhang et al., 1995). The level of reduced glutathione (GSH) was assayed by the method described by Hissin and Hilf (1973). The levels of endogenous redox enzymes viz. catalase (CAT), superoxide dismutase (SOD), glutathione peroxidase (GPx), and glutathione reductase (GR) were assessed as per the standard methods (Ghosh et al., 2010). The degree of DNA fragmentation in the selected tissues was measured by the diphenylamine reaction as described by Lin et al. (1997). DNA oxidation was assessed by RP-HPLC and was denoted as the ratio of 8-OHdG to 2-dG (Bolner et al., 2011). Intracellular ATP concentration was estimated using the commercially available assay kit (Abcam, Cambridge, MA, USA). Aldose reductase activity was measured following the method described by Nishinaka and Yabe-Nishimura (2001). The sorbitol dehydrogenase activity was measured following the protocol of Ulrich (1974). Glyoxalase-I activity was measured in accordance to McLellan and Thornalley (1989). The intracellular ATP concentrations were screened by commercial kits (Abcam, Cambridge, MA, USA). Levels of collagen IV in the tissue homogenates were determined using ELISA kits (R&D Systems, Inc., USA) according to the manufacturer’s guidelines.

### Western Blotting of Signal Proteins in Myocardial Tissues

The protein samples of hearts of specific cellular components namely cytosolic and nuclear fractions were separated following standard sequential fractionation process as described by Baghirova et al. (2015) and were quantified by ELISA (Bio-Rad, USA). The protein samples (20 μg) were subjected to 10% SDS-PAGE gel electrophoresis and western blotted as described by Dua et al. (2015a). The expressions of phospho-NF-κB (Ser 536), phospho-IκBα (Ser 32), IκBα, PKC β, PKC ε, PKC δ, and PARP were studied. The immunoblots were detected by 3,3′-diaminobenzidine tetrahydrochloride (Bangalore Genei, India). Normalization of protein expression was done by using GAPDH as loading control.

### Histological Assessment

Excised hearts of rats were immediately fixed in formalin and were subjected for paraffin blocking followed by sectioning. Sections of ∼5 μm thickness were stained with hematoxylin and eosin (H & E) to assess under microscope (Dua et al., 2015b). Masson Trichrome (MT) staining was performed following the protocol of Xiao et al. (2015). Finally, the sections were mounted with resinous mounting medium for microscopic observations.

### Statistical Analysis

The experimental data were interpreted by one-way ANOVA and expressed as mean ± SD followed by Dunnett’s *t*-test using computerized GraphPad InStat (version 3.05), GraphPad software, USA. The significance was considered when *p* < 0.05.

### *In Silico* ADME Prediction of Protocatechuic Acid

*In silico* ADME properties of the protocatechuic acid have been performed by the QikProp module in the Maestro Schrodinger (MS) software (Schrödinger, LLC, 2014). Here, ADME properties have been checked through Lipinski’s rule of five along with some important properties such as mol_MW, FOSA, FISA, PISA, donarHB, accptHB, percent human oral absorption, rtvFG, CNS, QPlogPo/w, QPlogHERG, and QPPCaco (Ray et al., 2015).

### Molecular Docking

The possible interaction patterns of protocatechuic acid with many receptors were investigated. To perform this work, the target proteins such as PKC-δ (PDB: 1PTR), PKC-β (PDB: 2I0E), IRS (PDB: 2Z8C), PI3K (PDB: 3DBS), Akt (PDB: 3D0E), AMPK (PDB: 4QFR), PARP (PDB: 5DS3), and NF-κB (PDB: 1A3Q) were retrieved from the PDB (Protein data bank, 2015). Protocatechuic acid, as ligand, was taken from the pubchem database. The preparation of protein structures to complete fault like missing loops, steric clashes, and missing atom names were performed by Protein Preparation Wizard. The ligand, protocatechuic acid, has been prepared in Schrödinger, LLC (2014). The grid was generated to locate the active sites of the proteins on the basis of co-crystal ligand attached with the crystal proteins. Finally, the SP docking studies (Schrödinger, LLC, 2014) were performed in Glide. For getting the amino acids which were mainly responsible for the ligand’s activity, the docking interactions were compared with the interactions present in the co-crystal ligand available in the PDB site.

## Results

### Effects on Fasting Blood Glucose Level, Body Mass Gain, Foods and Water Intake

**FIGURE 1:**
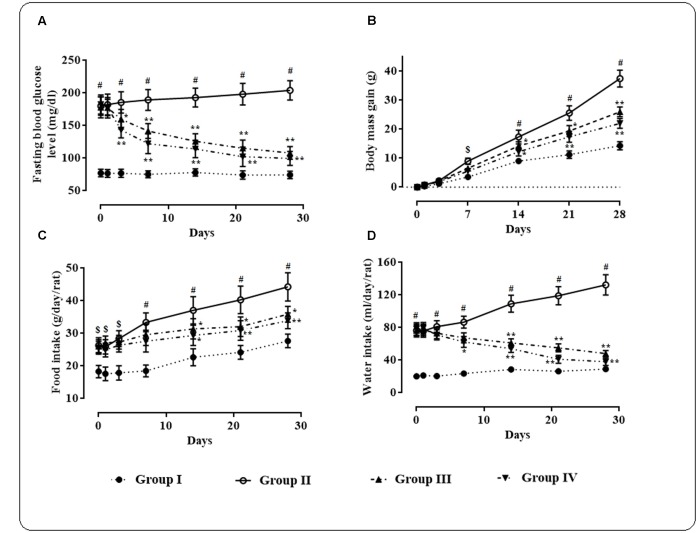
**Effects of protocatechuic acid on fasting blood glucose level** **(A)**, body mass gain **(B)**, food intake **(C),** and water intake **(D)** in T2D rats. Data were expressed as mean ± SD (*n* = 6). ^$^*p* < 0.01 compared with Group I; ^#^*p* < 0.01 compared with Group I; ^∗^*p* < 0.05 compared with Group II; ^∗∗^*p* < 0.01 compared with Group II. Group I: normal control; Group II: T2D control; Group III: T2D rats treated with protocatechuic acid (50 mg/kg, p.o.), Group IV: T2D rats treated with protocatechuic acid (100 mg/kg, p.o.).

High fat fed rats treated with small i.p. dose of streptozotocin exhibited significantly (*p* < 0.01) high fasting blood glucose level (170 ± 30 mg/dl) when compared with normal rats (**Figure 1A**).

Protocatechuic acid (50 and 100 mg/kg) treatment significantly alleviated (*p* < 0.05–0.01) fasting blood glucose level day 3 onward of therapeutic regime. However, maximum therapeutic efficacy was observed on 28th day of treatment with the reduction of ∼39.32 (*p* < 0.01) and 45.39% (*p* < 0.01) of fasting blood glucose at the doses of 50 and 100 mg/kg, respectively. The effects of protocatechuic acid on body mass gain, food and water consumption by the experimental rats of different groups were depicted in **Figures 1B–D**. In this study, the T2D rats exhibited significant (*p* < 0.05–0.01) increase in body mass gain when compared with normal rats. However, protocatechuic acid (50 and 100 mg/kg) treatment could significantly reduce body mass gain day 14 onward of the therapeutic regime. In this study, the T2D rats exhibited significant raise in food (*p* < 0.05–0.01) and water (*p* < 0.01) consumption when compared with normal rats. However, protocatechuic acid (50 and 100 mg/kg) treatment significantly reversed food (*p* < 0.05–0.01) and water (*p* < 0.01) consumption day 14 onward of therapeutic regime.

### Effects on Serum Insulin Level, HOMA-IR and HOMA-β

**FIGURE 2:**
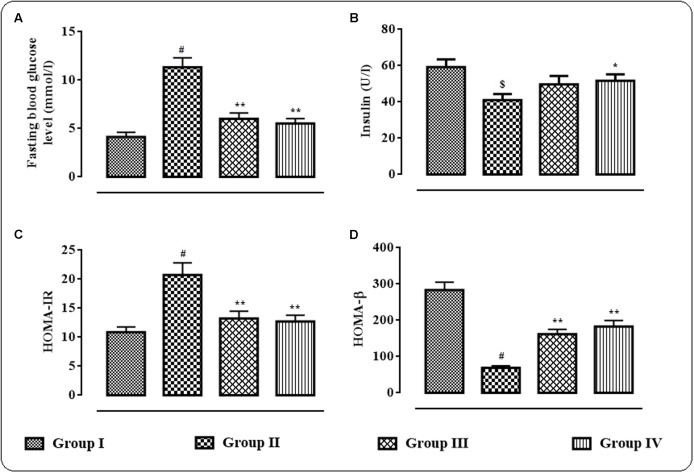
**Effects of protocatechuic acid on fasting blood glucose level (mmol/l)** **(A)**, serum insulin (U/l) **(B)**, HOMA-IR, **(C)** score and HOMA-β **(D)** score on day 29 of post-treatment in T2D rats. Data were expressed as mean ± SD (*n* = 6). ^$^*p* < 0.01 compared with Group I; ^#^*p* < 0.01 compared with Group I; ^∗^*p* < 0.05 compared with Group II; ^∗∗^*p* < 0.01 compared with Group II. Group I: normal control; Group II: T2D control; Group III: T2D rats treated with protocatechuic acid (50 mg/kg, p.o.), Group IV: T2D rats treated with protocatechuic acid (100 mg/kg, p.o.). HOMA-IR = (Fasting serum insulin in U/l × Fasting blood glucose in mmol/l)/22.5; HOMA-β = (Fasting serum insulin in U/l × 20/Fasting blood glucose in mmol/l) – 3.5.

The effects of protocatechuic acid (50 and 100 mg/kg) on fasting blood glucose level (mmol/l), serum insulin (U/l), HOMA-IR and HOMA-β have been shown in **Figures 2A–D**. T2D rats exhibited significantly (*p* < 0.01) high fasting blood glucose (mmol/l) on day 29, while, serum insulin level was significantly (*p* < 0.05) reduced on day 29 when compared with normal rats (**Figure 2B**). However, protocatechuic acid (50 and 100 mg/kg) treatment could significantly reverse the blood glucose (*p* < 0.01) levels to near normal status. On other hand, protocatechuic acid (100 mg/kg) could significantly (*p* < 0.05) improve insulin level in the sera of T2D rats. In this study, T2D rats exhibited significantly high (*p* < 0.01) HOMA-IR score (**Figure 2C**) with concomitant reduction (*p* < 0.01) of HOMA- β score when compared with normal rats (**Figure 2D**). Aforementioned alteration in fasting blood glucose level, HOMA-IR and HOMA- β scores indicated the establishment of hyperglycemia coupled with insulin resistance. On other hand, protocatechuic acid (50 and 100 mg/kg) treatment could significantly reverse HOMA-IR (*p* < 0.01) and HOMA-β (*p* < 0.01) scores near to normalcy (**Figures 2C,D**).

### Effects on Serum Biochemical Parameters

In this study, the effects of protocatechuic acid on different biochemical parameters were studied (**Table 1**). Significant elevation in the levels of total cholesterol (*p* < 0.01), triglycerides (*p* < 0.01) and LDL-cholesterol (*p* < 0.01) was observed in the sera of T2D rats. However, HDL-cholesterol (*p* < 0.01) level was significantly reduced in the sera of T2D rats. On other hand, protocatechuic acid (50 and 100 mg/kg) treatment could significantly reverse serum lipid profile (*p* < 0.01) of T2D rats near to normalcy. T2D rats exhibited a significantly (*p* < 0.01) high levels of glycosylated-hemoglobin, C-reactive proteins, LDH, CK, AGEs, and troponins in the sera. However, protocatechuic acid (50 and 100 mg/kg) treatment could significantly reverse glycosylated-hemoglobin, C-reactive proteins, LDH, CK, AGEs, troponin I and troponin II levels in the sera of T2D rats.

**Table 1:** Effects of protocatechuic acid on serum biochemical parameters of experimental rats.

Parameters	Group I	Group II	Group III	Group IV
Total cholesterol (mg/dl)	87.21 ± 7.33	164.21 ± 15.08^#^	136.74 ± 12.89^∗∗^	132.15 ± 13.54^∗∗^
HDL cholesterol (mg/dl)	34.50 ± 2.92	15.18 ± 1.67^#^	26.92 ± 2.11^∗∗^	27.89 ± 2.37^∗∗^
LDL cholesterol (mg/dl)	28.13 ± 2.33	108.72 ± 9.42^#^	76.93 ± 6.81^∗∗^	73.06 ± 7.18^∗∗^
Triglycerides (mg/dl)	122.91 ± 10.11	201.55 ± 19.98^#^	164.43 ± 13.17^∗∗^	156.02 ± 14.12^∗∗^
Glyco-hemoglobin (mg/g hemoglobin)	10.32 ± 0.08	0.71 ± 0.08^#^	0.48 ± 0.03^∗∗^	0.47 ± 0.08^∗∗^
LDH (U/l)	156.72 ± 14.82	272.54 ± 24.50^#^	197.33 ± 16.48^∗∗^	182.11 ± 14.75^∗∗^
CK (IU/mg of protein)	12.07 ± 1.20	18.05 ± 1.62^#^	15.59 ± 1.37^∗^	13.18 ± 1.02^∗∗^
C-reactive protein (mg/dl)	1.46 ± 0.15	2.88 ± 0.47^#^	1.84 ± 0.33^∗∗^	1.73 ± 0.24^∗∗^
AGEs (μg/ml)	415.48 ± 32.11	772.23 ± 60.19^#^	587.01 ± 55.18^∗∗^	528.44 ± 48.82^∗∗^
Troponin I (ng/ml)	1.33 ± 0.18	2.78 ± 0.54^#^	2.12 ± 0.24^∗^	1.97 ± 0.35^∗∗^
Troponin II (ng/ml)	472.76 ± 45.29	796.01 ± 73.67^#^	645.00 ± 54.12^∗^	622.71 ± 60.13^∗∗^


*Data were expressed as mean ± SD (*n* = 6). ^#^*p* < 0.01 compared with Group I; ^∗^*p* < 0.05 compared with Group II; ^∗∗^*p* < 0.01 compared with Group II. Group I: Normal control; Group II: T2D control, Group III: T2D rats treated with protocatechuic acid (50 mg/kg, p.o.), Group IV: T2D rats treated with protocatechuic acid (100 mg/kg, p.o.).*

### Effects on Signal Proteins in the Skeletal Muscle

**FIGURE 3:**
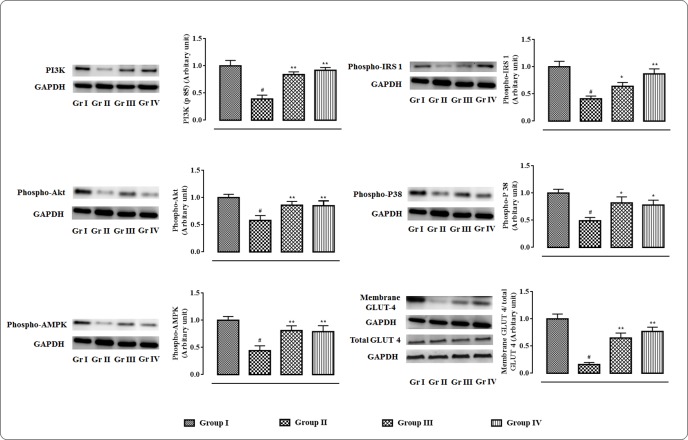
**Effects of protocatechuic acid in the expressions of signal proteins viz.** PI3K, IRS 1, Akt, P 38, AMPK in skeletal muscle of T2D rats followed by densitometric analysis of the respective protein levels and the normal control band was given an arbitrary value of 1. GAPDH was used as loading control. Data were expressed as mean ± SD (*n* = 6). ^#^*p* < 0.01 compared with Group I; ^∗^*p* < 0.05 compared with Group II; ^∗∗^*p* < 0.01 compared with Group II. Group I: normal control; Group II: T2D control; Group III: T2D rats treated with protocatechuic acid (50 mg/kg, p.o.), Group IV: T2D rats treated with protocatechuic acid (100 mg/kg, p.o.).

The western blot analyses of the signal proteins involved in glucose utilization in the skeletal muscle were performed (**Figure 3**). In this study, significant (*p* < 0.01) down-regulation in the expression of PI3K (p 85) was observed in skeletal muscle of T2D rats. On other hand, protocatechuic acid (50 and 100 mg/kg) treatment could significantly (*p* < 0.01) up-regulate PI3K (p 85) expression in the skeletal muscle of T2D rats. Phosphorylation of IRS 1 protein was significantly down-regulated resulting a significantly (*p* < 0.01) low phospho-IRS 1 expression in the skeletal muscle of T2D rats. However, protocatechuic acid (50 and 100 mg/kg) treatment could significantly (*p* < 0.01) reverse phospho-IRS 1 expression to near normal status. A significant reduction (*p* < 0.01) in the expression of phospho-Akt in the skeletal muscle of T2D rats indicated inactivation of Akt signaling. However, protocatechuic acid (50 and 100 mg/kg) treatment could significantly (*p* < 0.01) promote Akt signaling pathway via up-regulation of phosphorylation of Akt protein in the skeletal muscle of T2D rats. In this study, a significant (*p* < 0.01) down-regulation in the expression of membrane associated GLUT4 was observed in the skeletal muscle of T2D rats. However, protocatechuic acid (50 and 100 mg/kg) treatment could significantly (*p* < 0.01) up-regulate the expression of membrane associated GLUT4 in the skeletal muscle of T2D rats. In this study, T2D rats exhibited a significant (*p* < 0.01) down-regulation in the expressions of phospho-AMPK and phospho-P38 in the skeletal muscle. However, protocatechuic acid (50 and 100 mg/kg) treatment could significantly up-regulate (*p* < 0.01) the expressions of phospho-AMPK and phospho-P38 in the skeletal muscle of T2D rats.

### Effects on Polyol Enzymes, ATP and Collagenase IV in the Myocardial Tissue

**FIGURE 4:**
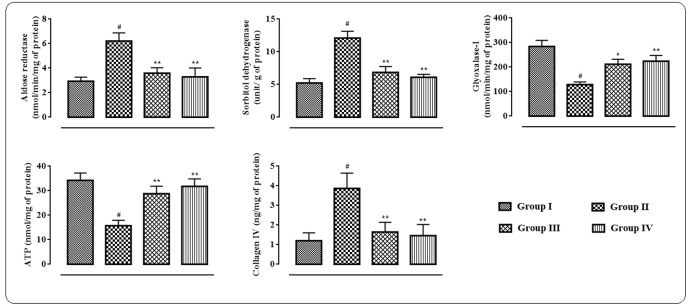
**Effects of protocatechuic acid on aldose reductase, sorbitol dehydrogenase, glyoxalase-I, ATP and collagen IV levels in myocardial tissues of T2D rats.** Data were expressed as mean ± SD (*n* = 6). ^#^*p* < 0.01 compared with Group I; ^∗^*p* < 0.05 compared with Group II; ^∗∗^*p* < 0.01 compared with Group II. Group I: normal control; Group II: T2D control; Group III: T2D rats treated with protocatechuic acid (50 mg/kg, p.o.), Group IV: T2D rats treated with protocatechuic acid (100 mg/kg, p.o.).

In this study, significant up-regulation in the levels of aldose reductase (*p* < 0.01) and sorbitol dehydrogenase (*p* < 0.01) with concomitant depletion of glyoxalase-I (*p* < 0.01) were observed in the myocardial tissue homogenate of T2D rats (**Figure 4**). However, protocatechuic acid (50 and 100 mg/kg) treatment could significantly down-regulate the levels of aldose reductase (*p* < 0.01), sorbitol dehydrogenase (*p* < 0.01) in the heart of T2D rats. On other hand, glyoxalase-I (*p* < 0.05–0.01) level was significantly improved in the myocardial tissue of T2D rats following protocatechuic acid (50 and 100 mg/kg) treatment. In this study, ATP level in the myocardial tissue homogenate was significantly (*p* < 0.01) reduced in T2D rats when compared to that of normal rats (**Figure 4**). However, treatment with protocatechuic acid (50 and 100 mg/kg) could significantly (*p* < 0.05–0.01) enhance intracellular ATP content in the myocardial tissues of T2D rats. In this study, T2D rats exhibited a significant (*p* < 0.01) elevation in the levels of collagen IV in the myocardial tissue (**Figure 4**). However, protocatechuic acid (50 and 100 mg/kg) treatment could significantly reverse collagen IV (*p* < 0.01) levels in hearts of T2D rats.

### Effects on Redox Status within Myocardial Tissues

**FIGURE 5:**
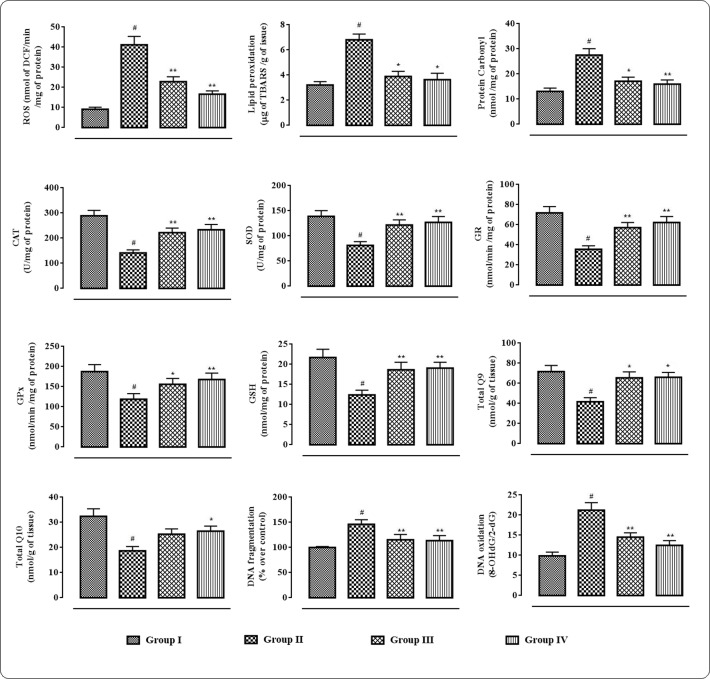
**Effects of protocatechuic acid on redox status viz.** ROS production, lipid peroxidation, protein carbonylation, SOD, CAT, GR, GPx, GSH, co-enzyme Q9, co-enzyme Q10, DNA fragmentation, DNA oxydation in myocardial tissues of T2D rats. Data were expressed as mean ± SD (*n* = 6). ^#^*p* < 0.01 compared with Group I; ^∗^*p* < 0.05 compared with Group II; ^∗∗^*p* < 0.01 compared with Group II. Group I: normal control; Group II: T2D control; Group III: T2D rats treated with protocatechuic acid (50 mg/kg, p.o.), Group IV: T2D rats treated with protocatechuic acid (100 mg/kg, p.o.). CAT unit, ‘U’, is defined as μmoles of H_2_O_2_ consumed per minute. SOD unit, ‘U’, is defined as the μmoles inhibition of NBT reduction per minute.

In this study, the extent/levels of intracellular ROS production, lipid peroxidation, protein carbonylation, endogenous anti-oxidant enzymes, GSH, co-enzymes Q, DNA fragmentation, and DNA oxidation in the cardiac tissues were depicted in **Figure 5**. In this study, T2D rats exhibited significantly high (*p* < 0.01) levels of intercellular ROS in the myocardial tissue. The levels of TBARS and carbonylated proteins were significantly (*p* < 0.01) amplified in the cardiac tissues of T2D rats. However, protocatechuic acid (50 and 100 mg/kg) treatment could significantly alleviate ROS production (*p* < 0.01), lipid peroxidation (*p* < 0.05), and protein carbonylation (*p* < 0.05–0.01) in the myocardial tissues of T2D rats. The levels of endogenous antioxidant enzymes viz. CAT, SOD, GPx, and GR and antioxidant metabolite viz. GSH were significantly (*p <* 0.01) decreased in the myocardial tissues of T2D rats when compared with normal rats. However, treatment with protocatechuic acid (50 and 100 mg/kg) significantly (*p <* 0.05–0.01) improved CAT, SOD, GST, GR, and GSH levels in the cardiac tissues of T2D rats. T2D rats exhibited significantly (*p* < 0.01) decreased levels of co-enzyme Q9 and Q10 in the cardiac tissue. However, protocatechuic acid (100 mg/kg) treatment could significantly reverse co-enzyme Q9 and Q10 in the cardiac tissue of T2D rats. In current investigation, the extents of fragmentation and oxidation of DNA were significantly increased in the cardiac tissue of T2D rats. However, Protocatechuic acid (50 and 100 mg/kg) treatment significantly (*p <* 0.01) reversed the DNA fragmentation and oxidation in the cardiac tissues of T2D rats.

### Effects on Pro-inflammatory Cytokines Levels

**FIGURE 6:**
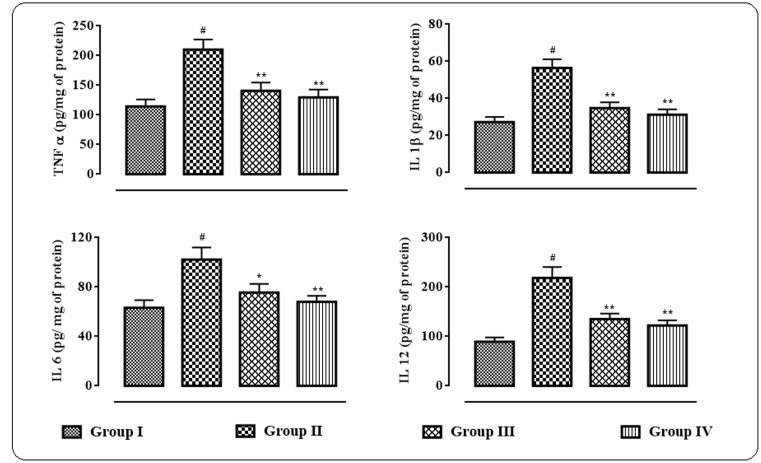
**Effects of protocatechuic acid on inflammatory biomarkers viz.** TNF α, IL 1β, IL 6, and IL 12 in the sera of T2D rats. Data were expressed as mean ± SD (*n* = 6). ^#^*p* < 0.01 compared with Group I; ^∗^*p* < 0.05 compared with Group II; ^∗∗^*p* < 0.01 compared with Group II. Group I: normal control; Group II: T2D control; Group III: T2D rats treated with protocatechuic acid (50 mg/kg, p.o.), Group IV: T2D rats treated with protocatechuic acid (100 mg/kg, p.o.).

The effects of protocatechuic acid on the inflammatory bio-markers in the sera were shown in **Figure 6**. In this study, significant (*p* < 0.01) elevation in the levels of IL 1β, IL 6, IL 12, and TNF α in the sera of T2D rats were observed. However, protocatechuic acid (50 and 100 mg/kg) could significantly reversed IL 1β (*p* < 0.01), IL 6 (*p* < 0.01), IL 12 (*p* < 0.05–0.01), and TNF α (*p* < 0.01) levels in the sera of T2D rats to near normal status.

### Effects on Signal Proteins in Hearts

**FIGURE 7:**
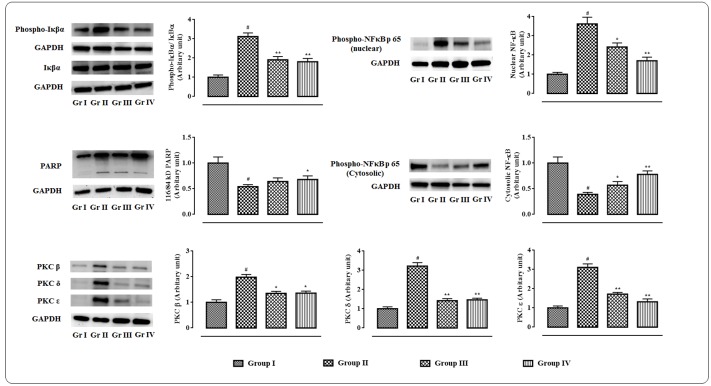
**Effects of protocatechuic acid on the expressions of signal proteins viz.** PARP, PKCs, IκBα, NF-κB involved in the inflammatory patho-physiology in myocardial tissues of T2D rats followed by densitometric analysis of the respective protein levels and the normal control band was given an arbitrary value of 1. GAPDH was used as a loading control. Data were expressed as mean ± SD (*n* = 6). ^#^*p* < 0.01 compared with Group I; ^∗^*p* < 0.05 compared with Group II; ^∗∗^*p* < 0.01 compared with Group II. Group I: normal control; Group II: T2D control; Group III: T2D rats treated with protocatechuic acid (50 mg/kg, p.o.), Group IV: T2D rats treated with protocatechuic acid (100 mg/kg, p.o.).

The western blot analyses of the signal proteins involved in inflammatory process in myocardial tissue were performed (**Figure 7**). In this study, significant (*p* < 0.01) up-regulation in the expressions of PKC isoforms was visible in the cardiac tissue of T2D rats. However, protocatechuic acid (50 and 100 mg/kg) could significantly down-regulate the expressions of PKC β (*p* < 0.05), PKC δ (*p* < 0.01), PKC ε (*p* < 0.01) in the cardiac tissue of T2D rats when compared with T2D control rats. T2D rats exhibited significant (*p* < 0.01) cleavage of PARP into cleaved form (84 kDa) from its full length (116 kDa) in the myocardial tissue. However, protocatechuic acid (100 mg/kg) treatment significantly (*p* < 0.05) attenuated PARP cleavage. Immunoblot analysis revealed that significant (*p* < 0.01) degradation of IκBα via its phosphorylation in the myocardial tissue of T2D rats. On other hand, protocatechuic acid (50 and 100 mg/kg) treatment could significantly (*p* < 0.01) reverse IκBα phosphorylation. In this study, significant (*p* < 0.01) up-regulation in the expression of NF-κB (p 65) in the nuclear fraction with concomitant down-regulation (*p* < 0.01) in the expression of cytosolic NF-κB (p 65) were observed in the myocardial tissues of T2D rats. The aforementioned observations suggested the nuclear translocation of NF-κB protein. However, protocatechuic acid (50 and 100 mg/kg) treatment could significantly reverse the expressions of nuclear and cytosolic NF-κB and thereby prevented the nuclear translocation of NF-κB protein.

### Histology of Heart Sections

**FIGURE 8:**
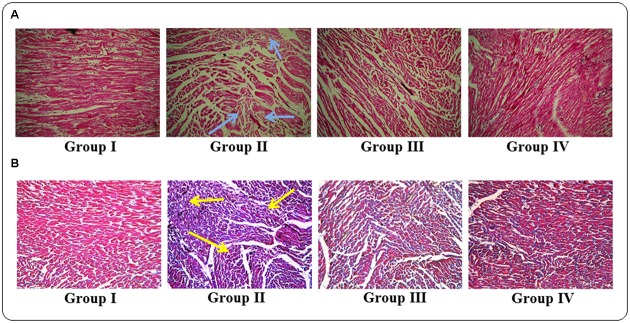
**Histological assessments of hearts of normal (Group I), T2D rats (Group II), protocatechuic acid (50 mg/kg, p.o.) treated T2D rats (Group III) and protocatechuic acid (100 mg/kg, p.o.) treated T2D rats following H & E** **(A)** and MT staining **(B)**. The H & E stained sections of heart (x 100) of T2D rats exhibited the irregular radiating pattern with injured interstitial tissues (blue arrows). MT staining showed collagen deposition in the hearts of T2D rats (yellow arrows). Protocatechuic acid (50 and 100 mg/kg) treatment significantly restored the radiating pattern of myocardial tissue. On other hand, protocatechuic acid (50 and 100 mg/kg) treatment significantly reduced blue stained portion indicating reduction of collagen deposition.

The histological sections of hearts of rats under different treatments were depicted in **Figure 8**. The H & E stained heart sections (x 100) of T2D rats exhibited the irregular radiating pattern with injured interstitial tissues (blue arrows). However, protocatechuic acid (50 and 100 mg/kg) treatment could significantly reduce the T2DM mediated histological abnormality and restored the muscle radiating pattern near to normalcy. MT staining of T2D rats indicated enhanced deposition of collagen (yellow arrows). Protocatechuic acid (50 and 100 mg/kg) treatment significantly reduced of blue stained portion, which is an indication of reduction of collagen deposition.

### *In Silico* ADME Observations

The ADME related descriptors were reported in the **Table 2**. Protocatechuic acid passes all the descriptors related to drug-likeness character of a compound. The QPlogHERG and CNS descriptor suggested that, protocatechuic acid may not produce toxic manifestation to the heart and central nervous system.

**Table 2:** The ADME results for the protocatechuic acid.

S. no.	Descriptors	Predicted values for protocatechuic acid
1	rtvFG	0
2	CNS	-1
3	mol_MW	154.122
4	FOSA	0
5	FISA	179.531
6	PISA	84.549
7	Donor HB	3
8	Accpt HB	3.5
9	QPlogPo/w	-0.445
10	QPlogHERG	-0.785
11	QPPCaco	49.774
12	Percent human oral absorption	54.714
13	Rule of five	0


### Molecular Docking Analysis

**FIGURE 9:**
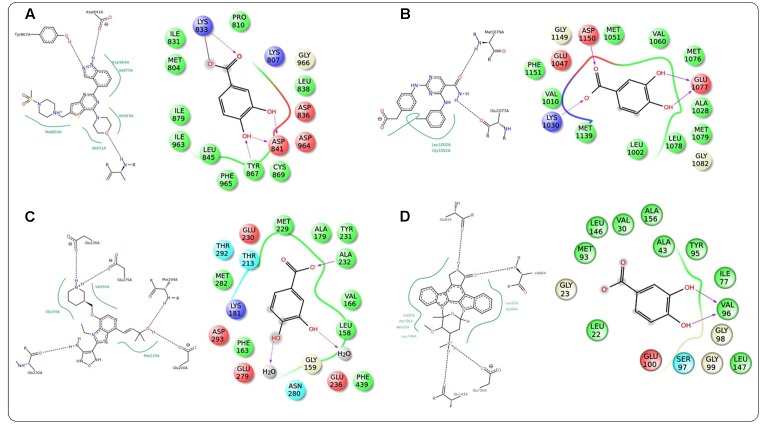
**Docking interactions of co-crystal ligand with the PI3K protein (PDB: 3DBS) and protocatechuic acid with corresponding amino acid residues of PI3K, respectively** **(A)**. Docking interactions of co-crystal ligand with the IRS protein (PDB: 2Z8C) and protocatechuic acid with corresponding amino acid residues of IRS, respectively **(B)**. Docking interactions of co-crystal ligand with the Akt protein (PDB: 3D0E) and protocatechuic acid with corresponding amino acid residues of Akt, respectively **(C)**. Docking interactions of co-crystal ligand with the AMPK protein (PDB: 4QFR) and protocatechuic acid with corresponding amino acid residues of AMPK, respectively **(D)**.

**FIGURE 10:**
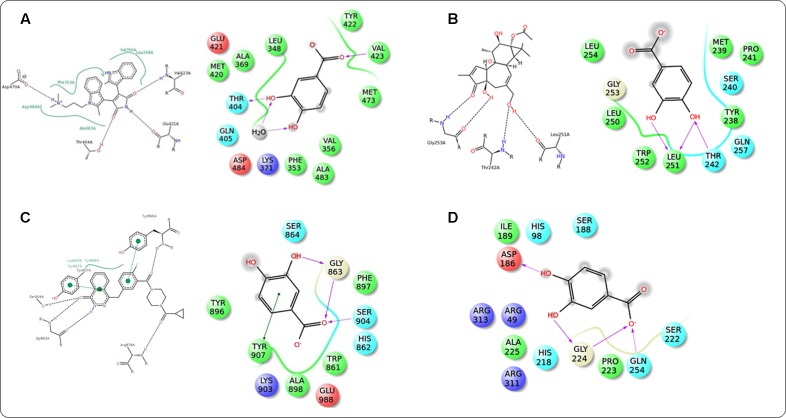
**Docking interactions of co-crystal ligand with the PKC β protein (PDB: 2I0E) and protocatechuic acid with corresponding amino acid residues of PKC β, respectively **(A)**.** Docking interactions of co-crystal ligand with the PKCδ protein (PDB: 1PTR) and protocatechuic acid with corresponding amino acid residues of PKCδ **(B)**. Docking interactions of co-crystal ligand with the PARP protein (PDB: 5DS3) and protocatechuic acid with corresponding amino acid residues of PARP **(C)**. Docking interactions of protocatechuic acid with the NF-κB protein (PDB: 1A3Q) **(D)**.

In this study, it was attempted to explore some possible key residues of the receptors which are especially responsible for the interactions with the protocatechuic acid. Dockings of the signal proteins responsible in glucose transport in the skeletal muscle were shown in **Figure 9**. In case of PI3K (PDB: 3DBS), three possible H-bond interactions were found with Asp 841 and Tyr 867 along with some hydrophobic interactions with Met 804, Ile 831, Ile 879, etc. (**Figure 9A**). The probable binding interactions of IRS (PDB: 2Z8C) exhibited two H-bonds with Glu 1077 and one important hydrophobic interactions with Leu 1002 (**Figure 9B**). Some different interactions were found with the Akt (PDB: 3D0E), such as H-bond interactions with Ala 232 and two co-crystal water molecules, however, the hydrophobic interactions (Met 229 and Val 166, etc.) were as similar as mentioned in the PDB file (**Figure 9C**). Another protein AMPK (PDB: 4QFR) produces similar two H-bonds with Val 96 and hydrophobic interactions with Leu 22, Leu 146, Met 93, Ala 156, etc. (**Figure 9D**). Dockings of the signal proteins responsible in inflammatory pathophysiology in the kidneys were shown in **Figure 10**. Two H-bond interactions were produced with the catalytic residue (Thr 404 and Val 423) of the protein PKC-β (PDB: 2I0E) and also some hydrophobic interactions were shown with Val 356, Leu 348, Ala 483, etc. (**Figure 10A**). Three H-bond interactions were predicted with most important residues Leu 251 and Thr 242 after binding with PKC-δ (PDB: 1PTR). Other many green ball shaped amino acids were mainly involved for neighboring hydrophobic interactions of the active cavity site (**Figure 10B**). The protein, PARP (PDB: 5DS3), exhibited three H-bond interactions with Gly 863 and Ser 904 along with some hydrophobic interactions with Tyr 896, Tyr 907, etc. Also here a similar π–π stacking interaction was prominently seen with Tyr 907 (**Figure 10C**). The protein NF-κB (PDB: 1A3Q) prominently showed three H-bond interactions with Gln 254, Gly 224, and Asp 186 along with two hydrophobic interactions with Ala 225 and Pro 223 (**Figure 10D**).

## Discussion

T2DM is rapidly becoming a forthcoming epidemic. In spite of several therapeutic strategies were proposed, T2DM and its associated pathogenesis remain increasingly uncontrolled. Persistent hyperglycemia, hyperlipidemia, increased ROS production and myocardial inflammation induce DC via alterations in downstream transcription factors, myocardial substrate utilization, myocyte growth, endothelial function, and myocardial compliance (Bhattacharjee et al., 2016b; Riaz et al., 2016). Persistent hyperglycemia exerts its injurious effects through a sequence of secondary transducers. One of the principle abnormalities is the excess generation of AGEs which deactivate NO and thereby, weaken coronary vasodilation (Singh et al., 2001; Riaz et al., 2016). Sustained hyperglycemia ensures excess generation of ROS which directly activate redox sensitive signaling cascade and participate in DC (Singh et al., 2001; Khanra et al., 2015). An increase in ROS with concomitant decrease in NO levels induces myocardial inflammation and endothelial dysfunction via PARP cleavage (Soriano et al., 2001). ROS also promote NF-κB signaling and thereby induce inflammation (Bhattacharjee et al., 2016a). Persistent hyperglycemia further promotes the activation of PKC signaling cascade in myocardial tissue (Way et al., 2001). The aforementioned toxicological events during T2DM integrally participate in the development and progression of cardiovascular complications to the diabetic patients. Considering the molecular basis of DC, an agent possessing hypoglycemic, anti-oxidant and anti-inflammatory activities would serve as a better therapeutic agent to counteract with DC.

Lowering the blood glucose level is principle approach to control DM and its associated toxic manifestations. In this study, T2DM was experimentally induced to Wister rats by feeding high fat diet following a single small dose of streptozotocin (35 mg/kg, i.p.) (Bhattacharjee et al., 2016b). Low dose of streptozotocin ensured partial damage of pancreatic β cell population while high fat diet resulted insulin resistance to the experimental rats. A significant decrease in serum insulin demonstrated the partial destruction of pancreatic β-cells. Significantly low HOMA-β score with significantly high HOMA-IR ensured the induction of insulin resistance to the rats (Reed et al., 2000). The aforementioned observations ensured the establishment of T2DM to the experimental rats. In this study, protocatechuic acid treatment significantly reduced fasting blood glucose level near to normalcy. The glycemic control ensured the significant drop in the level of glyco-hemoglobin and hyperglycemia mediated augmented ROS generation and thereby lowering the risk of DC (Khanra et al., 2015).

Skeletal muscle participates important role in the regulation of blood glucose level via its utilization/metabolism (Li et al., 2015; Zheng et al., 2015). In search of antihyperglycemic mechanism of protocatechuic acid, the signaling pathways involved in glucose metabolism within the skeletal muscle of T2D rats were investigated. PI3K pathway is an important signaling pathway of glucose utilization systems (Han et al., 2015). Activation of PI3K and Akt are the important steps in insulin action (Li et al., 2015). Akt is an important mediator of the glucose uptake process in the skeletal muscle. Tyrosine phosphorylation of IRS1 (Tyr 895) causes activation of PI3K which further promotes phosphorylation of Akt (Ser 473). Activation of aforementioned signaling cascade further promotes GLUT4 translocation. In case of T2DM, IRS1/PI3K/Akt/GLUT4 signaling is down-regulated. However, Protocatechuic acid treatment significantly increased the IRS1 phosphorylation, PI3K (p85) expression, Akt phosphorylation, and GLUT4 expression in T2D rats. AMPK regulates cellular energy homeostasis in glucose utilization process (Thakkar et al., 2015). Protocatechuic acid significantly activated AMPK phosphorylation (Thr 172) and thereby regulates cellular energy homeostasis in glucose utilization in the T2D rats. Activation of P 38 via phosphorylation also play important role in glucose transport and utilization (Macko et al., 2008). In this study, protocatechuic acid could significantly up-regulate the phosphorylation of P38. These results suggest that stimulation of IRS1/PI3K/AKT/AMPK/P 38/GLUT4 signaling pathway by protocatechuic acid in skeletal muscle would have significant role in its antihyperglycemic effect.

T2DM is associated with hyperlipidemia which largely contributes in cell death and thus to cardiac dysfunctions (Boudina and Abel, 2010). In this study, significantly elevated levels of membrane bound enzymes, LDH and CK, vindicated the cellular damage in T2D rats. High levels of serum lipids further promoted the deposition of cholesterol and triglycerides to the myocardial tissues, which is directly influencing the cardiac toxicological consequences (Boudina and Abel, 2010). Protocatechuic acid treatment could significantly reverse serum lipid level near to normalcy and thereby attenuate DC.

Oxidative stress plays critical role in the development of DC. The mechanisms of ROS production in diabetic hearts are yet to be clearly understood. However, earlier reports revealed that increased oxidative stress would be correlated with lipid overload, suggesting a role for fatty acid in the generation of ROS (Boudina and Abel, 2010). Hyperglycemia-induced formation of AGEs has also been regarded as the important source of oxidative free radicals (Pal et al., 2014).

In this study, a significantly high ROS level was observed in the myocardial tissues of T2D rats. The excess of ROS further promotes upsurges in lipid peroxidation, protein carbonylation with concomitant diminution of antioxidant enzymes in the myocardial tissues of T2D rats. However, protocatechuic acid treatment could significantly scavenge ROS level in the myocardial tissues resulting protection against oxidative damages of lipids and proteins. Protocatechuic acid also could up-regulate endogenous antioxidant molecules and thereby attenuate oxidative stress. T2D rats exhibited significant low level of GSH in the myocardial tissue, which indicated the over-utilization of GSH in a redox challenged cyto-environment. The redox challenged cellular environment further promotes oxidative damage and fragmentation DNA and causes cell death. In this study, cardiac tissue of T2D rats exhibited significant DNA fragmentation and oxidation. However, protocatechuic acid treatment could significantly attenuate DNA fragmentation and oxidation. The aforementioned prophylactic effect of protocatechuic acid may be due to its radical scavenging and antioxidant effect.

Persistent hyperglycemia leads to activation of polyol pathway which results excess production of AGEs in T2DM (Singh et al., 2014). The interactions between receptors for AGEs result a series of cellular events viz. oxidative stress, inflammation, extracellular matrix accumulation, etc., which further led to myocardial dysfunctions (Bodiga et al., 2014). In this study, protocatechuic acid significantly inhibited AGEs level in sera of T2D rats. Protocatechuic acid treatment inhibited the activation of polyol pathway by reducing the activities of aldose reductase and sorbitol dehydrogenase and increasing the activity of glyoxalase I. The reduction of AGEs production in protocatechuic acid treated T2D rats would be correlated with its effect on polyol enzymes.

Hyperglycemia-induced low grade myocardial inflammation have significant role in the development of DC. The increased level of C-reactive protein in the sera of T2D rats indicated the establishment of inflammation (Khanra et al., 2015). Besides, T2D rats exhibited significantly higher levels of TNF α, IL 1β, IL 6, and IL 12 in the sera of T2D rats. However, treatment with protocatechuic acid significantly reduced the levels of aforementioned inflammatory markers in the sera. In search of molecular mechanism, the immunoblot analyses were performed with the myocardial proteins. Activation of NF-κB signaling pathways play an important role in the inflammatory pathophysiology (Khanra et al., 2017). Intracellular oxidative stress promotes PARP cleavage, which further activates NF-κB signaling (Bhattacharya et al., 2013). On other hand, hyperglycemic redox stress also activates PKC through polyol activation (Ahmad et al., 2005). PKC activation also contributes in NF-κB activation in redox challenged cyto-environment. PKCs contribute in the accumulation of collagen and cause fibrosis (Bhattacharjee et al., 2016b). NF-κB is activated during inflammation by its phosphorylation and degradation of its inhibitor-κB (IκBα) via phosphorylation (Bhattacharjee et al., 2016a). Then phospho-NF-κB translocates to nucleus and activates the genes encoding inflammatory markers (Khanra et al., 2017). In this study, significant translocation of phospho-NF-κB (p 65) to nucleus from cytosol was observed in the myocardial tissue of T2D rats. However, protocatechuic acid could significantly attenuate inflammation in the myocardial tissue via inhibition of PKC/PARP/NF-κB signaling.

The histological assessment revealed that, irregular radiating pattern of cardiac muscle with injured interstitial tissues and collagen deposition in the heart of T2D rats. However, protocatechuic acid treatment could significantly attenuate DC visualized in the heart sections of protocatechuic acid treated T2D rats.

In ADME studies, Lipinski’s rule of five justified the drug-likeness character of protocatechuic acid (Veber et al., 2002). The rule suggested that the drug-like molecule must comply with the following five conditions: (i) log *p-*value must be between 0.4 and +5.6; (ii) molar refractivity must be between 40 and 130; (iii) molecular weight should be between 180 and 500; (iv) The number of atoms must be between 20 and 70 including H-bond donors and acceptors; and (v) The polar surface area must not be greater than 140 Å^2^ and/or fewer than 10 rotatable bonds. In earlier studies we have taken different classes of three standard marketed drugs, like aspirin, paracetamol, and rosiglitazone, checked their rule of five values in Maestro, and they all produce the acceptable value of 0 (maximum acceptable value is 4) (Ray et al., 2015). In this study, protocatechuic acid satisfied aforementioned Lipinski’s rule of five value reflecting the good *in silico* pharmacokinetic profile and thereby reflected the drug-likeness nature of protocatechuic acid. Finally, *in silico* molecular docking revealed the probable interactions of protocatechuic acid with PKC β, PKC-δ, NF-κB, PARP, PI3K, IRS, Akt, and AMPK. Protocatechuic acid had been predicted to offer H bonding and hydrophobic interactions within the active sites of these proteins and thereby regulate their role to control hyperglycemia and DN.

**FIGURE 11:**
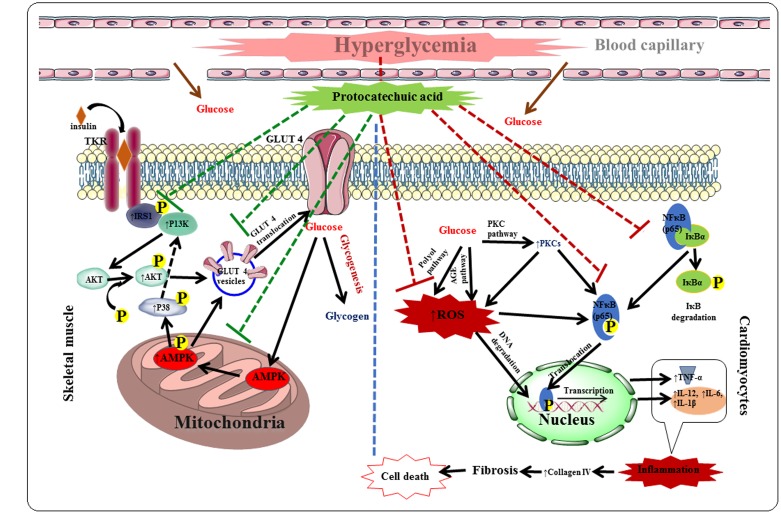
**The hypothesis developed regarding possible mechanism of protocatechuic acid in the management of DC.** Red dotted lines represent inhibition and green dotted line indicates activation.

Protocatechuic acid exhibited protective effect against DC via hypoglycemic, insulin-sensitizing, anti-oxidant and anti-inflammatory effects in T2D rats. The hypoglycemic and insulin-sensitizing effects would be mediated by stimulation of IRS1/PI3K/AKT/AMPK/GLUT4/P 38 signaling pathway in the skeletal muscle, while, anti-inflammatory effects would be documented with the inhabitation of PARP/PKC/NF-κB signaling cascade in the myocardial tissue. Protocatechuic acid also exerted significant antioxidant and radical scavenging effect in the myocardial tissue of T2D rats, which would be due to its multiple phenolic -OH groups within the molecule. The probable protective mechanism of protocatechuic acid has been depicted in **Figure 11**. Molecular docking analysis predicted the probable interactions within the active sites of signal proteins. ADME prediction revealed that, protocatechuic acid supports the drug-likeness character apparent from Lipinski’s rule of five. Therefore, protocatechuic acid would have a good possibility to be a new therapeutic agent for DC in future.

## Author Contributions

SD: designed the experiments, supervised and participated entire work. NB, RK, and TD: performed animal studies, biochemical analysis and phytochemical analysis. NB and SJ: performed western blot analysis. NB, SJ, and TD: performed histological analysis. AS and AN: performed *in silico* analysis. SD, SJ, and VDF: contributed in data analysis. SD and VDF: participated in writing the manuscript. All authors read and approved the final manuscript.

## Conflict of Interest Statement

The authors declare that the research was conducted in the absence of any commercial or financial relationships that could be construed as a potential conflict of interest.
